# How Well Do We Handle the Sample Preparation, FT-ICR Mass Spectrometry Analysis, and Data Treatment of Atmospheric Waters?

**DOI:** 10.3390/molecules27227796

**Published:** 2022-11-12

**Authors:** Lucas Pailler, Pascal Renard, Edith Nicol, Laurent Deguillaume, Angelica Bianco

**Affiliations:** 1 Laboratoire de Météorologie Physique, UMR 6016, CNRS, Université Clermont Auvergne, 63178 Clermont-Ferrand, France; 2 Laboratoire de Chimie Moléculaire, UMR 9168, CNRS, Ecole Polytechnique, 91128 Paris, France; 3 Observatoire de Physique du Globe de Clermont-Ferrand, UMS 833, CNRS, Université Clermont Auvergne, 63178 Clermont-Ferrand, France

**Keywords:** cloud water, cloud chemistry, molecular formula assignment, ultrahigh resolution mass spectrometry, environmental complex mixtures, solid phase extraction, lyophilization

## Abstract

FT-ICR MS (Fourier-transform ion cyclotron resonance mass spectrometry) analysis has shown great potential to aid in the understanding of the extremely high molecular diversity of cloud water samples. The main goal of this work was to determine the differences in terms of formula assignment for analytical (i.e., measurement replicates) and experimental replicates of a given cloud water sample. The experimental replicates, obtained by solid phase extraction, were also compared to the results obtained for freeze-dried samples to evaluate whether the presence of salts interferes with the analysis. Two S/N ratios, generally adopted for atmospheric samples, were evaluated, and three different algorithms were used for assignment: DataAnalysis 5.3 (Bruker), Composer (Sierra Analytics), and MFAssignR (Chemical Advanced Resolution Methods Lab). In contrast to other works, we wanted to treat this comparison from the point of view of users, who usually must deal with a simple list of *m/z* ratios and intensity with limited access to the mass spectrum characteristics. The aim of this study was to establish a methodology for the treatment of atmospheric aqueous samples in light of the comparison of three different software programs, to enhance the possibility of data comparison within samples.

## 1. Introduction

Atmospheric aerosol is composed of diverse chemical species that influence its role in atmospheric processes. It contains inorganics together with a very complex mixture of organic compounds [1,2,3]. The concentration of inorganic constituents, such as sulfate, nitrate, ammonium, and sea salt, is relatively well known, while the information available on the organic fraction is far less detailed [4,5,6,7]. This is mainly because of its complexity, which is linked to the multiple sources and to the efficient transformation of organic compounds in the atmosphere [8]. Moreover, the concentration of individual organic compounds is often very low [5].

In cloud water, this intricacy moves up a notch. Cloud droplets contain a complex mixture of water-soluble organic matter (WSOM) originating from the scavenging of soluble gases and the dissolution of aerosol particles [9,10]. During the cloud’s lifetime, aqueous-phase reactions lead to (photo-)oxidative transformations that potentially produce small oxidized organic compounds [11,12,13,14] and high-molecular-weight organic matter [15,16]. Moreover, cloud WSOM is modified by microbial transformations [17,18]. Overall, the cloud WSOM composition is controlled by sources and is heavily processed, making the matrix difficult to unravel [19]. Historically, researchers began with targeted analytical methods to focus on specific classes of compounds, like short chain carboxylic acids, aldehydes, or more recently, amino acids [20,21,22,23,24,25]. Only recent developments in high resolution mass spectrometry have enabled the non-targeted analysis of the whole composition of cloud water WSOM [26,27,28,29,30].

Unfortunately, it is still difficult to explore the composition of cloud WSOM; in contrast to aerosol, cloud water is an aqueous medium whose concentration is dependent on the cloud’s microphysical properties (i.e., liquid water content, droplet radius) and is typically very diluted. Most of the time, clouds collect in the free troposphere and have WSOM concentrations below 15 mgC L^−1^ [23,31,32,33,34]. 

Non-targeted approaches relying on the ultrahigh resolving power of Fourier transform ion cyclotron resonance mass spectrometry (FT-ICR MS) have shown great potential to aid the understanding of the extremely high molecular diversity of cloud WSOM [26,27,28,30]. In particular, electrospray ionization (ESI), which is a soft ionization technique for hydrophilic compounds, has been widely used for WSOM in rainwater, atmospheric aerosol, fog, and cloud water [35,36,37,38,39,40,41,42]. The high resolution provided by FT-ICR MS analysis enables the assignment of elemental molecular formula (MF); multiple algorithms and software programs have been developed by instrument makers and users and associated with different interfaces and disparate levels of control on the assignment parameters [43].

The analysis of atmospheric matrices brings analytical challenges that have not been fully assessed, especially concerning sample preparation, ion production, data analysis, and interpretation. Moreover, the presence of inorganic salts may interfere with the analysis. Considering the low concentration of WSOM in atmospheric aqueous samples (cloud and fog), solid phase extraction (SPE) is generally employed to concentrate and desalt the organic matter [26,27,28]. However, it is tricky to evaluate the efficacy of the extraction and to gain information on the concentrations of compounds in the original sample, since the extraction efficiency is not equal for all compounds. Important contributions have been made in this field through the optimization of protocols for WSOM extraction and concentration, and also through the use of mass spectrometry analysis, elemental formula assignment, and compositional analysis [44,45]. Nevertheless, few studies have focused on the variability in the measurement system itself, the acceptable signal-to-noise (S/N) ratio in the data treatment, and the comparison of experimental replicates, especially concerning atmospheric matrices. It is particularly important to establish and verify these parameters to improve and facilitate sample comparison within datasets and across experiments [46].

The main goal of this study was to determine the differences in terms of formula assignment for analytical (i.e., measurement replicates) and experimental replicates (i.e., analysis of SPE replicates) from the same sample. We evaluated the use of two S/N ratios that are generally adopted for atmospheric samples and three different software programs for assignment: Bruker Compass DataAnalysis 5.3 (referred to as DataAnalysis) developed by Bruker; Composer 1.5.4 developed by Sierra Analytics; and the open-source software MFAssignR developed by Chemical Advanced Resolution Methods (ChARM) Lab. The first software program, DataAnalysis, is an instrumental software that is used for the internal recalibration and peak-list extraction of mass spectra; the second, Composer, is a licensed calculation method that was developed for elemental composition assignment in collaboration with the petroleum research community. The third software program, MFAssignR, is publicly available and it was designed for environmentally complex mixtures and has been tested on aerosol samples.

In contrast to other works, we wanted to assess this comparison from the point of view of users, who usually have limited access to the mass spectrum characteristics and, most of the time, must deal with a list of *m/z* ratios and intensities. Therefore, in this work, we compared the assignments obtained for three analytical and experimental replicates of the same cloud water sample. The experimental replicates, obtained by SPE, were also compared to the results obtained after freeze drying pre-concentration of the same cloud sample to evaluate whether the presence of salts interferes with the analysis. The goal of this study was to establish a methodology that can be used for the treatment of aqueous atmospheric samples based on a comparison of three different software programs to enhance the possibility of data comparison within samples.

## 2. Materials and Methods

### 2.1. Site and Cloud Sampling

Sampling was performed at the Puy de Dôme station (PUY) (45.77° N, 2.96° E, 1465 m a.s.l.) in the Massif Central region (France). PUY is part of the French national platform Cézeaux-Aulnat-Opme-puy de Dôme (CO-PDD) [47] and belongs to the following international networks: the European Monitoring and Evaluation Programme (EMEP), Global Atmosphere Watch (GAW), and Aerosols, Clouds, and Trace Gases Research Infrastructure (ACTRIS). The PUY summit is frequently under cloudy conditions, on average 30% of the year, with greater occurrences during winter and autumn than during spring and summer [47]. This makes PUY a reference site from which clouds can be sampled and studied [48].

One cloud water sample was collected at PUY on 8 October 2021 (6.40–10.10 am UTC). This sample had enough volume to perform all the analyses required for the assessment of the methodology. Moreover, it showed dissolved organic carbon and inorganic ions concentrations in line with cloud water samples of marine origin, which represented most of the samples collected at PUY [48].

Sampling was performed using the aluminum cloud water collector previously described under non-precipitating and nonfreezing conditions [48]. Before cloud collection, the impactor was cleaned using ultrapure (Milli-Q) water and sterilized by autoclaving. A sample blank was obtained by spreading autoclaved MilliQ water on the impactor just before sampling. Immediately after sampling, cloud and blank samples were filtered using 0.2 μm nylon filters (Fisherbrand™) to eliminate insoluble particles and microorganisms and then stored at −20 °C. The microphysical and physico-chemical characterization of the cloud water sample was performed for the observation service PUYCLOUD, which is available online at https://www.opgc.fr/data-center/public/data/puycloud (accessed on 20 September 2022). The results are reported in Table S1 of the Supplementary Materials.

### 2.2. Sample Treatment and Schema of the Experiment

The cloud water sample was thawed at ambient temperature (≈20 °C) in a bench hood. SPE was used to concentrate the cloud WSOM and remove the inorganic salts before the ESI FT-ICR MS analysis. Three experimental replicates were obtained for the comparison. The Strata-X (Phenomenex) cartridges (1 g of sorbent contained in TEFLON^®^ tubes) were used for SPE and were conditioned through consecutive application of 3 mL isopropanol, 6 mL acetonitrile, and 6 mL methanol containing 0.1% of formic acid and 6 mL of MilliQ water containing 0.1% of formic acid. TEFLON^®^ tubes were used to avoid contamination released by other plastic polymers with potential interferences in the mass spectrum. Fifty milliliters of cloud water with pH 4.5 was applied at a rate of 1 mL min^−1^ to the cartridge. The cartridges were then rinsed with 4 mL MilliQ acidified water to remove the inorganic salts. Cartridges were subsequently dried and analytes were eluted with 2.0 mL acetonitrile/methanol/ MilliQ (45/45/10) at pH 10.4 with NH_4_OH (28% in water) [26,49]. SPE extracts were stocked at 4 °C in brown glass vials with TEFLON^®^ cap until analysis, which was performed within 1 week after SPE. The sample blank was extracted by SPE using the same procedure used for the cloud water sample.

SPE is commonly used for atmospheric aqueous samples, but the retention of some compounds can be very low. For this reason, we tested another concentration method for the preservation of heat-sensitive materials—lyophilization (freeze-drying). Two 50 mL frozen aliquots of the cloud water sample and one 50 mL aliquot of the sample blank were lyophilized in 50 mL Falcon^®^ that had been previously rinsed with ethanol and MilliQ water, covered with Parafilm^®^ foil, and subjected to a Heto PowerDry LL3000 freeze-drier. After lyophilization (LYO), each extract was dissolved in 2 mL of a mix of acetonitrile/methanol/MilliQ (33/33/33). All solvents were of HPLC grade or higher.

**Figure 1:**
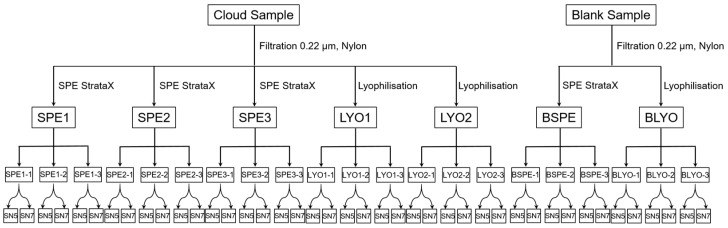
Schema of the experiment. SPE = solid phase extraction, LYO = lyophilization; BSPE = blank SPE; BLYO = blank LYO; SN = signal-to-noise ratio (S/N).

The sample treatment described produced three SPE extracts, named SPE1, SPE2, and SPE3; two LYO extracts, LYO1 and LYO2; and two blanks, one from SPE (BSPE) and one from LYO (BLYO). Seven different samples were thus obtained and analyzed separately by ESI(-) FT-ICR MS using the procedure described in Section 2.3. For more clarity, the schema of the experiment is depicted in Figure 1. Each sample was analyzed in triplicate by three consecutive injections in the ESI(-) FT-ICR MS, and the analytical replicates were named SPE1-1, SPE1-2, SPE1-3; SPE2-1, SPE2-2, etc., as reported in Figure 1. After internal recalibration, the signal was extracted using two S/N ratios of 5 and 7, as described in Section 2.4. At this step, we had 42 peak-lists that were assigned using the three software programs described in Section 2.5. We ended the experiment with 126 lists of molecular formulas (MFs).

The blank samples were treated as individual samples, and the MFs assigned to BSPE and BLYO were excluded from the samples as the last step of the data treatment, as reported in Section 3.5 of the Results and Discussion section.

### 2.3. ESI FT-ICR MS Analysis

The high-resolution mass spectrometry analysis was performed using a SolarixXR 9.4 T (Bruker, Germany), equipped with an electrospray ionization (ESI, Bruker) source, set in negative ionization mode. The instrument was externally calibrated with Tuning Mix from Agilent. Samples were infused directly into the ESI source. The parameters were optimized to obtain a stable ion current with a minima ion injecting time into the mass analyser. The infusion flow rate was 2.0 µL min^−1^, the drying gas temperature was 200 °C, and the drying and nebulizing gas flow rate were 4.0 L min^−1^ and 1 bar, respectively. The ESI capillary voltage was 3.9 kV. Three hundred scans were accumulated for each spectrum. Methanol was injected prior to the injection of each sample, and acquisition was performed to evaluate the potential presence of residual pollutants. The acquisition size was set to 8 M, resulting in a mass resolving power of up to (6.6 ± 1.6) × 10^5^ for the full mass range.

### 2.4. Preliminary Treatment with DataAnalysis

Each spectrum was preliminarily treated immediately after acquisition with DataAnalysis. Spectra were internally recalibrated using the recalibrant list reported in Table S2, which contains compounds that were selected after identifying their isotopic fine structures in mass spectra obtained from real recalibrated cloud samples. The linear recalibration was chosen, and the average standard deviation obtained after this step was below 0.1 ppm. After recalibration, the peak-list was extracted with two S/N ratios in order to evaluate which one was the most suitable for the analysis of atmospheric aqueous samples. In DataAnalysis, the noise was determined by considering the whole mass range of the spectrum.

The treatment with DataAnalysis allowed extraction of a peak-list from a recalibrated mass spectrum, along with the resolution for each peak and each intensity value.

### 2.5. Formula Assignment with Composer, DataAnalysis, and MFAssignR

#### 2.5.1. Composer

Composer has been widely used by researchers working on atmospheric organic aerosols [50,51,52,53] and on aquatic WSOM [54,55]. It has already been used for cloud water molecular characterization [18,26,27,49]. Composer has numerous features that act as comprehensive tools for internal mass recalibration and molecular formula assignment. The internal mass recalibration of cloud water samples was previously performed on Composer. Nevertheless, since the peak-lists extracted from DataAnalysis had already been internally recalibrated, in the present work Composer was only used for the molecular formula assignment. Similarly, the signal was not filtered by S/N ratio. For the molecular formula assignment, we searched for H loss producing ions with a single negative charge in the *m/z* range 100–1000 Da; no radicals were allowed. The Double Bond Equivalent (DBE) was set to be in the range 0–25 in accordance with Giannopoulos et al. [56] and Koch et al. [57], and the elemental composition was in the range C_1-70_H_2-140_O_1-25_N_0-4_S_0-1_ [27]. The *m/z* tolerance for the assignment was set to 0.5 ppm, and the DOM-NOM (dissolved organic matter—natural organic matter) rules were chosen for the attribution [58,59]. The Composer assignment relies on a de novo calculation (where de novo stands for the first of a series) based on the *m/z* value, set at 300 Da, the matching tolerance, and the elemental range constraint. CH_2_ and H_2_ molecular formula extensions were used to extrapolate from a de novo calculation to find target peaks related by these natural patterns. All parameters are reported in Table S3. Seven criteria were applied to exclude formulas that do not occur abundantly in natural organic matter: DBE must be an integer value, 0.2 ≤ H/C ≤ 2.4, O/C ≤ 1.5, N/C ≤ 0.5, S/C ≤ 0.2, 2 ≤ H ≤ (2C + 2), and 0 < O ≤ (C + 2). In the case of multiple formulas being assigned to the same peak, we considered the value with lower error in the assignment.

#### 2.5.2. DataAnalysis

In DataAnalysis, molecular formula assignment is performed by the SmartFormula tool, which automatically calculates possible molecular formulas for a selected mass range. DataAnalysis uses an algorithm that is based on the fact that the integer part and the fractional part of the molecular mass are linearly independent for organic molecules up to a molecular mass of about 1000 Da. That hypothesis only works for C, H, N, and O elements; the other elements are dealt with using a classic “try and error” method as they are present in smaller numbers at those masses. The molecular formula calculation is based on the isotopic masses, their abundances, and valences. For molecular formula assignment with DataAnalysis, we used the same parameters reported for the Composer assignment.

#### 2.5.3. MFAssignR

The MFAssignR package was designed to allow a comprehensive and transparent data processing for research applications involving environmentally complex mixtures and was applied, in particular, to atmospheric samples [43]. The MFAssignR package has a variety of functions, written in R programming language, for internal recalibration, MF assignment, S/N estimation, and isotopic filtering. Similar to the procedure described for the assignment with DataAnalysis and Composer, recalibration and S/N correction were not applied to the peak-list. Nevertheless, we decided to apply isotope filtering, which is a crucial step to avoid incorrect monoisotopic assignment. This function allows a tentative filtering of masses with ^13^C and ^34^S from the mass list and creates a separate mass list containing the monoisotopic masses along with the polyisotopic masses and their types (^13^C or ^34^S isotopes). Those lists were used as separate inputs in the MFAssignR molecular formula assignment. The molecular formula assignment was performed using the same parameters reported for Composer and DataAnalysis. Similar to Composer, MFAssignR uses the de novo calculation. The output of the function gives a list of ambiguous (multiple molecular formulas that have been assigned to the same peak) and unambiguous (molecular formulas that have been assigned to a unique mass) molecular formulas. Only MFs in the unambiguous list were considered for further data comparison since the list of ambiguous MFs was empty in most cases.

## 3. Results and Discussion

### 3.1. Analytical Replicates

**Figure 2:**
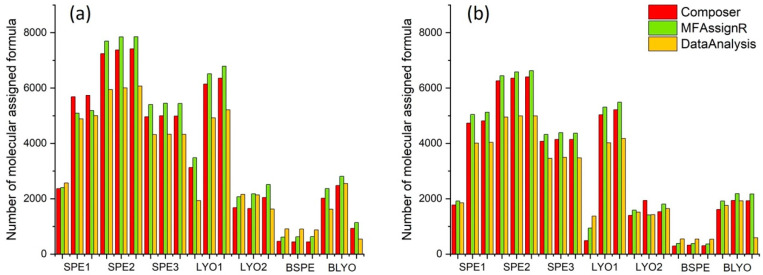
(**a**) Number of assigned molecular formulas for peak-lists extracted with S/N (signal to noise) 5 and (**b**) number of assigned molecular formulas for peak-lists extracted with S/N 7. Red bars indicate results obtained with Composer, green bars results obtained with MFAssignR, and yellow bars results obtained with DataAnalysis.

The first comparison performed focused on the number of MFs for the analytical replicates. Figure 2 depicts the number of MFs for each replicate and sample. As expected, the number of MFs was higher for S/N 5 than for S/N 7, except for the blank samples (BSPE and BLYO), which showed comparable values. Composer and MFAssignR tended to assign similar numbers of MFs, while DataAnalysis always attributed a lower number of MFs, except for the sample BSPE. A difference between replicates was detected: SPE1-1 had a lower number of MFs than SPE1-2 and SPE1-3, and the same discrepancy was observed for LYO1-1 compared with LYO1-2 and LYO1-3. On the other hand, SPE1-2 and SPE1-3 presented comparable numbers of MFs, as observed for LYO1-2 and LYO1-3, for the three software programs used. At this point, one should go back to the quality of the spectrum and try to find the reason for this difference; however, as an FT-ICR MS user, it is not always possible to have access to this information, and sometimes users can only rely on the peak-list. This result highlights the importance of the acquisition of replicates of the spectrum, and we suggest that at least three analytical replicates for each sample be used. Considering the good agreement between SPE1-2 and SPE1-3 and between LYO1-2 and LYO1-3, the replicates SPE1-1 and LYO1-1 were discarded.

We calculated the average and standard deviation for the number of MFs for each sample and software program used and we found that, for SPE, the standard deviation was always lower than 1.7% (S/N 5) and 1.5% (S/N 7), while for LYO it was higher. We observed very different signals for LYO1 and LYO2, but the replicates of LYO1 (standard deviation of 4.0% and 2.7% at S/N 5 and S/N 7, respectively) were more similar than for LYO2 (standard deviation of 15.1% and 17.4% at S/N 5 and S/N 7, respectively). This could be linked to the presence of salts in the freeze-dried extracts, which made the spectrum noisier. Moreover, the presence of salts can lead to interference in the internal recalibration and in the assignment [60]. For this reason, the present work is mostly focused on the SPE results.

We also checked the number of MFs in the blank samples. In our previous works, which involved assignment with Composer, the peak-list of the blank was excluded from the peak-list of the sample. However, it is not possible to carry out this procedure for all software programs, and it is not always accepted by the FT-ICR MS community [46]. For this reason, we decided to also assign MFs to the blank samples with the same procedure used for the SPE and LYO samples. At first glance, more MFs (up to 2368 and 2183 for S/N 5 and S/N 7, respectively) were attributed to BLYO compared with BSPE (up to 908 and 581 for S/N 5 and S/N 7, respectively). Moreover, in contrast to the other samples, DataAnalysis provided more MFs than Composer and MFAssignR.

In conclusion, we would like to highlight the need for analytical replicates, which are necessary to exclude instrumental variation. With the acquisition of three replicates, we were able to discard SPE1-1 and LYO1-1. After comparison, we merged the MF lists, considering only the common MFs and the intensity of the third replicate. We decided to consider the third replicate, because the mass signal was generally more stable.

**Figure 3:**
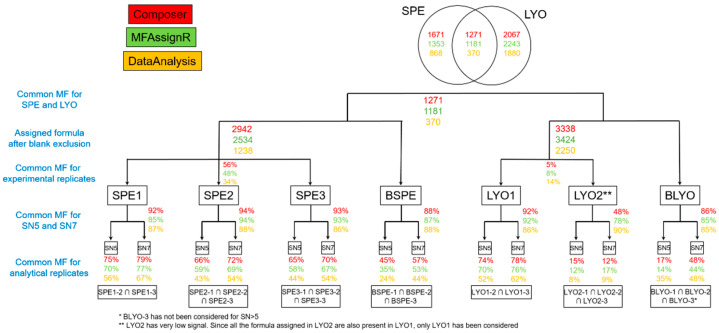
Percentage of MFs (molecular formulas) in common for each sample, considering only the number of MFs (percentage in number). Colors represent the data treatment applied for the three software programs considered in this work: red for Composer, green for MFAssignR, and yellow for DataAnalysis. The percentages in the first bottom line represent the common MFs for analytical replicates; those in the second line represent the common MFs for the S/N 5 (signal to noise) and S/N 7 ratios; and those in the third line represent the common MFs for the experimental replicates. The fourth line reports the number of MFs obtained after the exclusion of blank MFs, while the fifth line presents a comparison between SPE and LYO.

**Figure 4:**
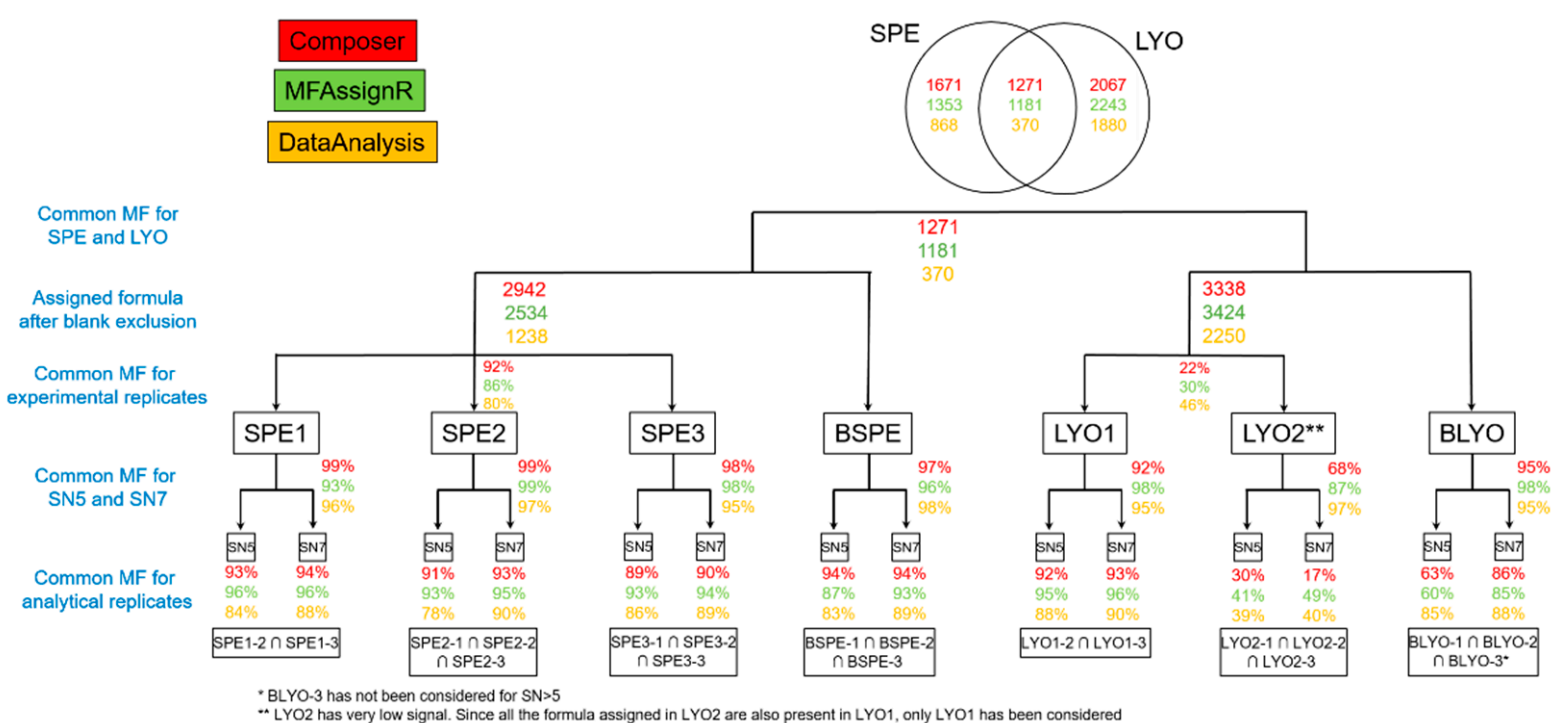
Percentage of MFs (molecular formulas) in common for each sample, considering only the number of MFs (weighted percentage). Colors represent the data treatment applied for the three software programs considered in this work: red for Composer, green for MFAssignR, and yellow for DataAnalysis. The percentages in the first bottom line represent the common MFs for analytical replicates; those in the second line represent the common MFs for the S/N 5 (signal to noise) and S/N 7 ratios; and those in the third line represent the common MFs for the experimental replicates. The fourth line reports the number of MFs obtained after the exclusion of blank MFs, while the fifth line presents a comparison between SPE and LYO.

Figure 3 and Figure 4 depict, in the bottom line, the percentage of common MFs for each sample, considering only the number of MFs (percentage in number, Figure 3) or the number of MFs and their intensities (weighted percentage, Figure 4). As expected, the number of common MFs at S/N 5 was always lower than at S/N 7. To avoid interference from the instrument background signal, it is probably more appropriate to use S/N 7 for data treatment. Moreover, there were generally more common MFs in Composer and MFAssignR than those assigned by DataAnalysis. We found that the common MFs represented 62 ± 18% of the total for Composer, 58 ± 19% for MFAssignR, and 49 ± 12% for DataAnalysis (LYO2 was not considered). It is very difficult to explain this result because the internal recalibration was the same for all peak-lists.

In response to this finding, we also calculated the weighted percentages of common MFs, taking into account the intensity of the signal. These calculations confirmed that the peaks with higher intensities were more likely to be assigned to the same MFs in the three replicates than the peaks with lower intensities. In Figure 4, the trends are the same as those observed for Figure 3: lower percentages for S/N 5 than for S/N 7 and comparable values for Composer and MFAssignR that are higher than those found for DataAnalysis. The weighted percentages of common MFs were 90 ± 9% for Composer, 90 ± 10% for MFAssignR, and 87 ± 4% for DataAnalysis (LYO2 was not considered). This confirms that the intensity of the mass signal should be considered when comparing MF datasets.

### 3.2. S/N Comparison—SPE

Considering the results presented in Section 3.1., filtering by S/N 7 gave a better match (>90% for intensity-weighted values) between the three replicates acquired for each sample. However, we observed that sometimes a few MFs were assigned only at S/N 7 and not at S/N 5; this is probably due to the loss of the signals of homologous series or isotopes, which generally had low intensities in the mass spectrum, at S/N 7, which led to the erroneous assignment. The number of MFs found only at S/N 7 was particularly high for the assignment with DataAnalysis, with an average of 208 MFs (<4.6%) assigned only at S/N 7, while for Composer and MFAssignR the number of MFs was mostly below 10 (<0.2%). To avoid incorrect assignment, we removed these MFs from the lists. In conclusion, when working with Composer and MFAssignR, in light of the low number of MFs found only at S/N 7, we did not need to compare the filtering at S/N 5 and S/N 7. Nevertheless, this difference was more important in DataAnalysis and probably needs to be considered.

### 3.3. Experimental Replicates—SPE

Figure 3 shows that only 56% of the MFs were present in the three SPE extracts, considering the results from Composer software, and this percentage decreased for MFAssignR (48%) and DataAnalysis (34%). Assuming similar ionization efficiencies, the use of intensity-weighted MFs may improve the comparison between samples. Looking at the weighted percentages, the values increased to 92%, 86%, and 80% for Composer, MFAssignR, and DataAnalysis, respectively. To better understand the source of this difference, some parameters, such as the average intensity, the MF error, the numbers of carbon, hydrogen, oxygen, nitrogen, and sulfur, and the DBE, were compared. Figures S1–S3 depict the differences in the DBE and number of atoms for each fraction following data treatment with Composer, MFAssignR, and DataAnalysis, respectively. As fractions, we defined the MFs common to the three SPEs (“Common”), present only in SPE1, SPE2, or SPE3 (“only SPE1”, “only SPE2”, and “only SPE3”, respectively), common to SPE1 and SPE2 (“SPE1_SPE2”), common to SPE1 and SPE3 (“SPE1_SPE3”), and common to SPE2 and SPE3 (“SPE2_SPE3”). We noticed that these parameters did not differ significantly between the fractions and, therefore, cannot explain the low reproducibility of the SPE process.

We also plotted a van Krevelen diagram, reporting the hydrogen to carbon ratio (H/C) as a function of the oxygen to carbon ratio (O/C) for each fraction and software program to exclude the hypothesis that compounds that are not in common have similar characteristics. However, the dots in the van Krevelen diagram were equally spread and did not group in specific regions.

**Figure 5:**
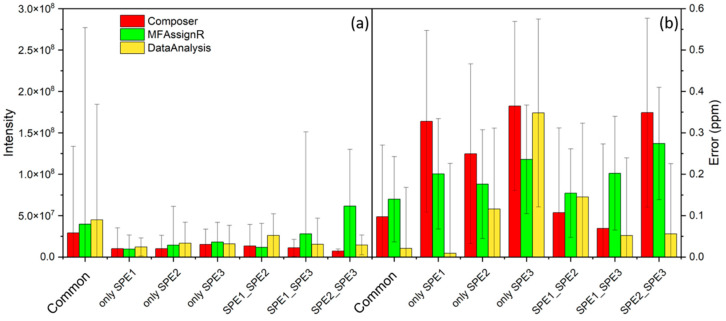
(**a**) Average intensity of the MFs (molecular formulas) common to the three SPE (“Common”); present only in SPE1, SPE2, or SPE3 (“only SPE1”, “only SPE2”, and “only SPE3”, respectively); common to SPE1 and SPE2 (“SPE1_SPE2”); common to SPE1 and SPE3 (“SPE1_SPE3”); and common to SPE2 and SPE3 (“SPE2_SPE3”). The error bars represent the standard deviations of the average intensities. The colors represent the software programs used for the data treatment: red for Composer, green for MFAssignR, and yellow for DataAnalysis. (**b**) Similar to (**a**), average errors (ppm) and standard deviations associated with the attribution of the MFs are given.

It is worth noting that compounds found only in one or two fractions generally had lower intensities than those common to the three SPEs, leading to the conclusion that the main difference in SPE was the loss of less concentrated compounds, which were not always well retained by the solid phase. Figure 5a shows the average intensity of the MFs in each fraction and for each software program, which was significantly lower for almost all fractions compared with “Common”. However, we also found that compounds common to SPE2 and SPE3 for data treatment with MFAssignR had higher intensities; this was because this fraction contained four MFs with intensities higher than 3 × 10^8^, which drove the intensities of all fractions. These four MFs corresponded to C_45_H_72_O_5_S, C_51_H_76_O_5_S, C_25_H_39_N_3_O_5_, and C_19_H_14_OS, and were found also by Composer and DataAnalysis in some fractions, as reported in Table S4. However, the error associated to the assignment, in particular regarding C_19_H_14_OS, led us to consider that the error in the assignment may be a source of discrepancies between the three SPEs. Figure 5b has a similar structure to Figure 5a and reports the error rate in the assignment for each fraction and software program. At first glance, the error was significantly higher than the values found for “Common” for the fractions “OnlySPE1”, “OnlySPE2”, “OnlySPE3”, and “SPE2_SPE3” and was comparable for the two remaining fractions.

As a conclusion to this comparison, the use of three SPE replicates for each sample and consideration of only the common MFs would be the best option to achieve a reliable assignment. However, this procedure, in addition to being time consuming, is also challenging to complete for atmospheric water samples, which have generally low sampling volumes. The use of one SPE remains more affordable, and in the future the error threshold during assignment should be decreased. However, it is difficult to increase the S/N threshold while avoiding the loss of information on less concentrated molecules. In order to lessen the importance of MFs with low abundance, which are subject to higher variability in the SPE process, the intensity of the peak along with the MF should be considered. For further comparison, only MFs in the list “Common” were considered.

### 3.4. S/N Comparison and Experimental Replicates—LYO

The two aliquots of the cloud water sample concentrated by LYO gave extremely different signals in the FT-ICR MS and, in the case of LYO2, extremely poor spectra. Concerning LYO1, the results obtained for the analytical replicates and the S/N comparison between S/N 5 and S/N 7 were similar to those obtained for SPE in terms of the number of MFs and the weighted number of MFs (Figure 2). Nevertheless, LYO2 gave completely different results and showed poor repeatability between analytical replicates, always below 50% for the three software programs. Additionally, the comparison between S/N 5 and S/N 7 was not satisfactory, with only the results from DataAnalysis being comparable to those obtained for SPE. For this reason, LYO2 was not considered for further comparisons, and only results from LYO1 were taken into account for the discussion.

### 3.5. Blank Exclusion

As reported in Section 3.1, in our previous works [18,26,29,49], we excluded the spectrum of the blank in the spectrum of the sample using the specific tool in Composer software. From the beginning, we preferred to exclude rather than subtract blank peaks, which may be tricky due to shifts in abundance and potential carryover from the sample to blanks if other samples are analyzed prior to a blank. However, the exclusion function is not available in all the software programs tested and, for this reason, we decided to treat the blank as a sample, assign MFs, and then exclude the MFs found in the blank from the list “Common”.

It is worth noting that from the internal recalibration of the spectrum of BSPE in DataAnalysis, we were able to find the same recalibrants as in the samples, and the results were equal for S/N 5 and S/N 7, giving an average standard deviation of 0.051 ± 0.004 ppm on the calibration slope, lower than 0.1 ppm, which is considered the threshold value for satisfactory internal recalibration. This means that some of the recalibrants were probably impurities contained in the solid phase that were eluted with the sample during the extraction process. This hypothesis was strengthened because the number of recalibrants found in BLYO was generally lower and at a low intensity, leading to non-optimal recalibration. The standard deviation associated with the recalibration was 0.34 ± 0.26. However, this result could have also been due to the large presence of double peaks detected in LYO.

The exclusion of the MFs of BSPE in the SPE data treatment list “Common” led to the elimination of 222 (7.0%), 244 (8.8%), and 230 (15.7%) MFs for Composer, MFAssignR, and DataAnalysis, respectively. These numbers increased drastically for the exclusion of MFs of BLYO in LYO1: 1121 (25.1%), 1135 (24.9%), and 603 (21.1%) MFs were excluded, respectively, for data treatment with Composer, MFAssignR, and DataAnalysis.

The methodology used for blank correction for direct infusion into an ESI source is still under debate in the mass spectrometry community [61], and it is still difficult to evaluate whether this correction is satisfactory or not. However, the procedure presented in this work seems to correlate with the complexity of the matrix and the pre-concentration process, leading to fewer MFs being excluded for more simple spectra (for SPE) and more MFs being excluded for more complex spectra (for LYO).

### 3.6. Comparison SPE vs. LYO

The next step in our work was the comparison between results from solid phase extraction (SPE) and freeze-drying (LYO). The data treatment presented in the previous sections showed that SPE is largely more repeatable than lyophilization. Nevertheless, the objective here is to compare these two methodologies in detail. We compared the MF list containing the MFs in common between SPE1, SPE2, and SPE3, with exclusion of the MFs from BSPE, to the MFs list obtained by the exclusion of MFs from BLYO to LYO1. Three fractions were identified: “Only SPE” means MFs found only in the SPE data treatment, “Only LYO” means MFs found only in the LYO data treatment, while “Common” refers to MFs found in both data treatment groups. In all cases, less than 50% of the MFs were in common between the SPE and the LYO process, in accordance with the results reported in Table 1. Moreover, the calculation of weighted percentages, considering the intensities associated with the MFs, did not give better results.

**Figure 6:**
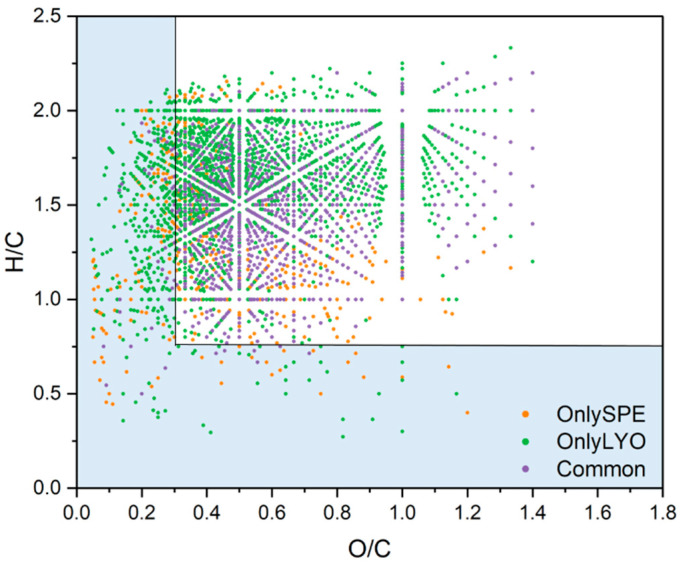
Van Krevelen diagram of the MFs in common between the LYO and SPE (“Common”) in violet, those only present in the SPE extract (“OnlySPE”) in orange, and those only present in the lyophilized extract (“OnlyLYO”) in green. Data treatment was conducted with MFAssignR.

The van Krevelen diagram may suggest whether SPE or LYO tended to result in the loss of a specific class of compounds. Figure 6 reports the van Krevelen diagram obtained for the three fractions of MFs in common between LYO and SPE, for those only attributed to the SPE extract, and for those only attributed to the LYO extract. At first glance, the number of green dots (“OnlyLYO”, 381 MFs) in the area with O/C ≤ 0.3 and H/C ≤ 0.75 (light blue area in Figure 6), corresponding to less oxidized compounds, is higher than those associated to the fractions “Common”, 47 MFs, and “OnlySPE”, 196 MFs. This could suggest that LYO is more appropriate for less oxidized compounds. However, the percentages in number of MFs reported for the considered values of O/C and H/C were similar for “OnlyLYO” (17.0%) and “OnlySPE” (14.5%), suggesting that the presence of more MFs in this range was simply due to the higher number of MFs in the “OnlyLYO” fraction (2243 MFs) than in the “OnlySPE” fraction (1353 MFs).

In conclusion, LYO pre-concentration is less reproducible and gives poor quality spectra compared with SPE. This is probably due to the higher concentration of salts in the LYO extracts, which prevents the efficient ionization of organic compounds or induces the formation of adducts, which were not investigated in this work. The results obtained are hardly comparable with the SPE results, and more research work is needed to improve the LYO procedure. Thus, the use of SPE for the pre-concentration of cloud water samples is recommended. 

### 3.7. Software Program Comparison

The last section of this work focused on the similarities and differences between the three software programs used for data treatment. As already explained in Section 1, FT-ICR MS instruments are frequently organized as national or international facilities with temporally limited access for the users. For this reason, we wanted to compare these software programs by only initializing them with simple peak-lists. This comparison considers only the SPE extraction.

**Figure 7:**
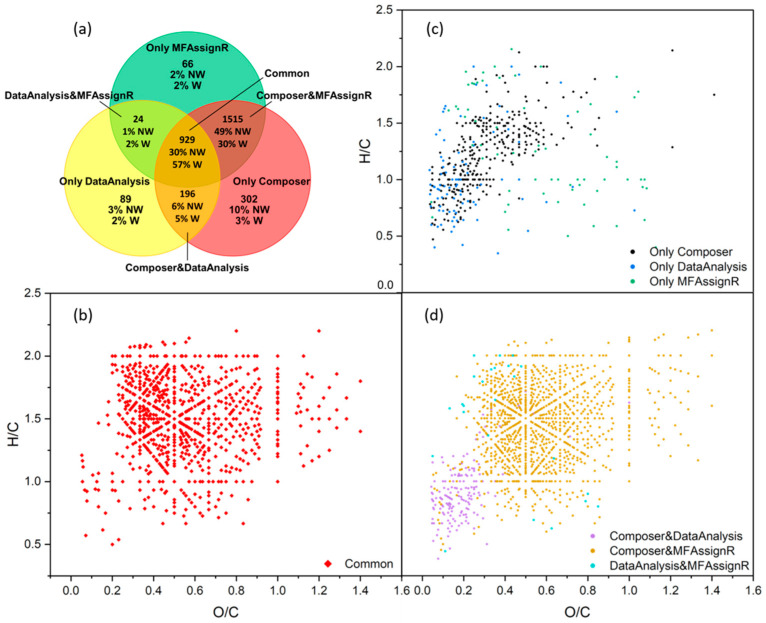
(**a**) Venn diagram comparing the number of MFs, the non-weighted (NW) and the weighted (W) percentages assigned by data treatment with Composer (red), MFAssignR (green), and DataAnalysis (yellow); (**b**) van Krevelen diagram of compounds belonging to “Common”; (**c**) van Krevelen diagram of compounds belonging to “Only Composer” (black), “Only DataAnalysis” (blue), and “Only MFAssignR” (green); (**d**) van Krevelen diagram of compounds belonging to “Composer&DataAnalysis” (violet), “Composer&MFAssignR” (ochre), and “DataAnalysis&MFAssignR” (turquoise).

**Figure 8:**
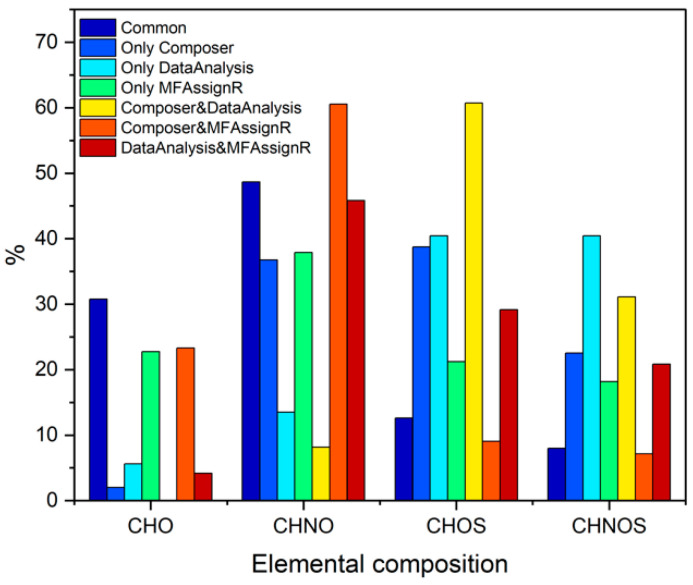
Comparison of the number of compounds (in percentage on the number of MF) containing carbon, hydrogen, and oxygen (CHO); carbon, hydrogen, nitrogen, and oxygen (CHNO); carbon, hydrogen, oxygen, and sulfur (CHOS); and all five elements (CHNOS) for the seven fractions of the Venn diagram shown in Figure 7a.

**Figure 9:**
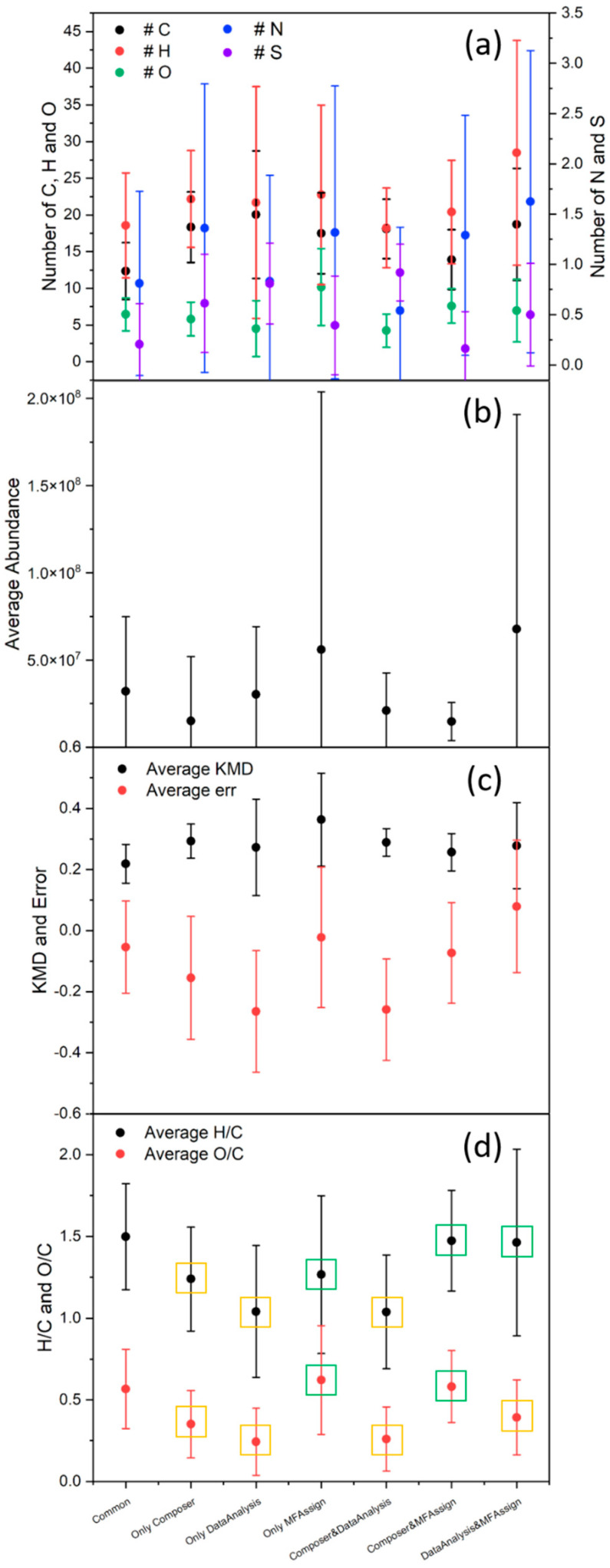
Comparison of (**a**) the number of atoms, (**b**) the average abundance, (**c**) the Kendrick mass defect (KMD) and error in the assignment, and (**d**) the H/C and O/C ratios for the seven fractions of the Venn diagram shown in Figure 6a. The error bars represent the standard deviations. In (**d**), the green square means that the value is not significantly different from “Common”, while the yellow square means that it is (*t*-test, 0.05 threshold value).

First of all, we compared the number of compounds obtained for each data treatment and the corresponding weighted and non-weighted percentages, as reported in Figure 7a. The number of MFs uniquely assigned by the three software programs was surprisingly low, even when the weighted percentage was considered (57%). A very large number of MFs (1515) were common between Composer and MFAssignR but were not attributed by DataAnalysis. To better compare the assignment performed with these software programs, similar to the fractions defined previously, we defined seven fractions: “Common” for compounds in common between the three software programs; “Composer&DataAnalysis”, “Composer&MFAssignR”, and “DataAnalysis&MFAssignR” to indicate the respective intersections of the Venn diagram; and “Only Composer”, “Only MFAssignR”, and “Only DataAnalysis” to indicate the groups of MFs assigned only by one software program. Many parameters, such as the elemental composition, the average abundance, the error, and the Kendrick mass defect (KMD), were compared and are reported in Figure 8 and Figure 9.

Considering Figure 8, the percentage of CHO compounds (i.e., those containing only carbon, hydrogen, and oxygen) in “Common” was significantly higher than that in the other fractions, suggesting a good assignment of this group of compounds overall. On the other hand, adding heteroatoms led to significant discrepancies in the assignment, especially regarding sulfur in CHOS and CHNOS. To better analyze this difference, as shown in Figure 9a, the number of atoms for each fraction was compared; no real trend was observed, and the results for the fraction “Common” were generally in line with those of the other fractions, even though the *t*-test (threshold value of 0.05) showed that all values were significantly different from those of “Common”. The comparison of the average abundance, the KMD, and the error associated with the assignment is presented in Figure 9 b and c. However, these parameters also did not show notable trends.

Lastly, we analyzed the O/C and H/C ratios, as reported in Figure 9d, and we noticed that all fractions except “Only MFAssignR” and “Composer&MFAssignR” were significantly different from “Common”, as reported with the green and yellow squares in Figure 9d. This led us to consider the van Krevelen diagram for each fraction, shown in Figure 6b–d. At first glance, the fraction “Composer&MFAssignR” contained an important number of MFs, confirmed by two software programs, which should not be discarded. On the other hand, the fraction “Composer&DataAnalysis” was located in a precise range of O/C and H/C, which also contained most of the MFs associated with the “Only Composer” and “Only DataAnalysis” fractions. The assignment in this part of the diagram is quite controversial, especially considering that most of the MFs in this range contain sulfur [62]. As reported in Section 2.5.2, the DataAnalysis algorithm uses the hypothesis that the integer part and the fractional part of the molecular mass are linearly independent for small organic molecules (<1000 Da), which is valid only for the CHO and CHNO compounds. MFs containing other heteroatoms were assigned using the “try and error” method, which is less reliable and can lead to systematic errors.

In conclusion, Composer and MFAssignR produced similar lists of MFs, while DataAnalysis provided a lower number of MFs. It is quite difficult to compare the three software programs since all parameters investigated showed similar values for all fractions. The only significant difference was in the O/C and H/C ratios, which was more visible in the van Krevelen diagram. A large number of MFs, spread all over the van Krevelen diagram, were confirmed only by Composer and MFAssignR and should not be discarded. A portion of the MFs assigned by Composer and DataAnalysis were located in a precise region and could be due to an error in the assignment linked to S attribution. To clarify this point, results from Composer and MFAssignR were directly compared, excluding DataAnalysis.

**Figure 10:**
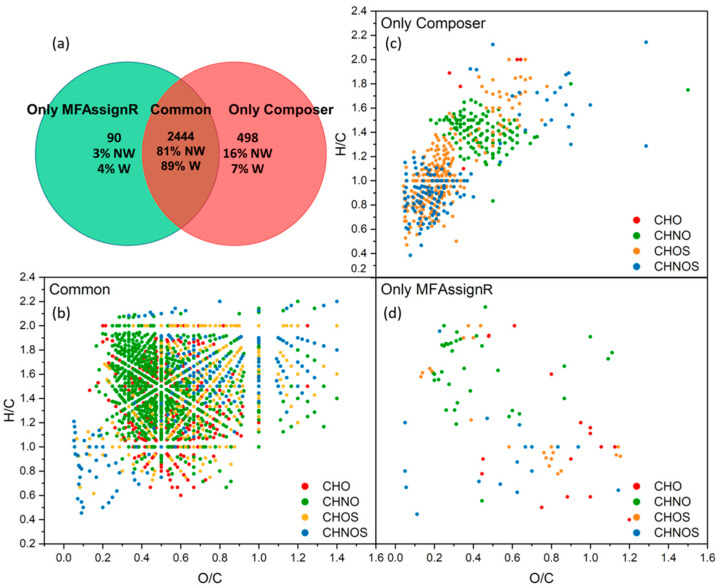
(**a**) Venn diagram comparing the number of MFs, the non-weighted (NW) and the weighted (W) percentages assigned by data treatment with Composer (red) and MFAssignR (green); (**b**) van Krevelen diagram of compounds belonging to “Common”: in red are compounds containing carbon, hydrogen, and oxygen (CHO), in green are compounds containing carbon, hydrogen, nitrogen, and oxygen (CHNO), in yellow are compounds containing carbon, hydrogen, oxygen, and sulfur (CHOS), and in blue are compounds containing carbon, hydrogen, nitrogen, oxygen, and sulfur (CHNOS); (**c**) van Krevelen diagram of compounds belonging to “Only Composer”; (**d**) van Krevelen diagram of compounds belonging to “Only MFAssignR”.

Similar to the fractions used in the previous paragraph, we defined three fractions, “Common”, “Only Composer”, and “Only MFAssignR”, in accordance with the Venn diagram presented in Figure 10a. First of all, we observed that the number of MFs in “Common” increased drastically, up to 89%, when we considered the weighted percentage, and the number of MFs in the fraction “Only MFAssignR” was below 5% when we considered both the weighted and non-weighted percentages. The van Krevelen diagrams, presented in Figure 10b–d, showed that MFs assigned only by MFAssignR were spread between the CHO, CHNO, CHOS, and CHNOS compounds and covered all of the H/C and O/C ratios, meaning that it would be difficult to find a systematic error for the assignment of these MFs.

We investigated the elemental composition, the average abundance, the KMD, and the error for the assignment, and we found that the abundance is slightly, but significantly, higher for the fraction “Only MFAssignR” (5.9 × 10^7^) than for the fraction “Common” (2.1 × 10^7^). This indicates that some of the highest abundant mass peaks were assigned differently by Composer and MFAssignR. On the other hand, the differences between “Common” and “Only Composer” were more evident: the percentage in number of sulfur-containing MFs in this fraction was four times higher than that in “Common”. Moreover, these compounds were all located in the same region in the van Krevelen diagram (Figure 10b), suggesting a potential systematic error in the assignment of sulfur, as already noticed for the comparison of the three software programs. We must consider that Composer was initially developed to meet the demands of the petroleomics community [63] and, thus, the parameters should be more carefully optimized to adapt to the needs of the atmospheric chemistry community [54]. Conversely, a special script was developed in MFAssignR to avoid the incorrect monoisotopic assignment of masses containing ^13^C or ^34^S, increasing the reliability of this software program for the assignment of S-containing compounds. Additionally, CHNO compounds seemed to be grouped in a specific range of O/C and H/C ratios, but it is difficult to speculate more on this group.

In light of these results, and considering that data treatment with two or three software programs is extremely time consuming, we would like to emphasize the good agreement between the Composer and MFAssignR results. The fact that only 90 MFs were assigned only by MFAssignR, and the evidence of a nonsystematic error, led us to conclude that MFAssignR is probably better adapted to our sample type. Thus, it will be used for assignment in our future works.

## 4. Conclusions

The disparities between the analytical and experimental replicates of the same cloud water sample were identified in this paper. Three distinct software packages for assignment and two signal-to-noise thresholds were examined. The goal of this study was to replicate the post-processing of peak-lists from the perspective of users with restricted access to the mass spectrum.

As a conclusion, we want to emphasize the need for analytical replicates in order to eliminate instrumental variability. We were able to exclude unusual spectra by collecting three replicates. Additionally, we found that a signal-to-noise threshold of seven allowed for more effective noise reduction without sacrificing the sample composition data. Only two S/N thresholds were compared. However, the S/N threshold could be variable in the mass spectrum, with lower values at lower masses and higher values at higher masses.

Three SPE replicates were carried out; this is the best option for a trustworthy task. However, this process is not only time-consuming, but it is also not optimal for atmospheric aqueous samples, which often are limited by small sampling volumes. For this reason, the use of one SPE remains more affordable. The analysis of the discrepancies between SPE replicates suggested a connection with the accepted error for the assignment. Additionally, the comparison of the experimental replicates revealed that the molecular formula should be considered along with the peak intensity to downplay the significance of chemical formulas with low abundance, which are more susceptible to variability in the SPE process.

Two extraction techniques were evaluated, and it is important to note that LYO pre-concentration both yields worse quality spectra than SPE and it is less repeatable. Unfortunately, the sample volume was not large enough to produce a third LYO experimental replicate, and this extraction process should be investigated and optimized in the future. In light of the results reported in the present work, we advise the use of SPE to concentrate cloud water samples.

Regarding the software package comparison, we would like to highlight the strong agreement between the Composer and MFAssignR results. Other software packages have been developed for MF assignment but were not compared in the present study. The performed comparison shows that MFAssignR is probably better suited to our type of samples, since it was particularly developed for environmental samples, and it will be used for assignment in our future works.

## Figures and Tables

**Table 1:** Weighted and non-weighted percentages obtained for the comparison of SPE and LYO; “SPE” means MFs found only in the SPE data treatment, “LYO” means MFs found only in the LYO data treatment, while “Common” refers to MFs found in both data treatment groups.

Software Program	Fraction	Percentage Non-Weighted	Percentage Weighted
Composer	SPE	26.6	23.2
	LYO	32.9	30.3
	Common	40.5	46.4
MFAssignR	SPE	22.7	24.4
	LYO	37.7	29.7
	Common	39.6	45.9
DataAnalysis	SPE	24.9	24.0
	LYO	53.9	45.1
	Common	21.2	30.9

## Data Availability

The data presented in this study are available in the Supplementary Materials.

## Supplementary Materials

The following supporting information can be downloaded at: https://www.mdpi.com/article/10.3390/molecules27227796/s1, Figure S1: Average DBE, number of carbon, hydrogen, nitrogen, oxygen and sulfur of the MF common to the three SPE (“Common”), present only in SPE1, SPE2 or SPE3 (“only SPE1”, “only SPE2”, and “only SPE3”, respectively), common to SPE1 and SPE2 (“SPE1_SPE2”), to SPE1 and SPE3 (“SPE1_SPE3”) or SPE2 and SPE3 “SPE2_SPE3”. The error bar represents the standard deviation of the average intensity. The MF assignment was performed with Composer software; Figure S2: Average DBE, number of carbon, hydrogen, nitrogen, oxygen and sulfur of the MF common to the three SPE (“Common”), present only in SPE1, SPE2 or SPE3 (“only SPE1”, “only SPE2”, and “only SPE3”, respectively), common to SPE1 and SPE2 (“SPE1_SPE2”), to SPE1 and SPE3 (“SPE1_SPE3”) or SPE2 and SPE3 “SPE2_SPE3”. The error bar represents the standard deviation of the average intensity. The MF assignment was performed with MFAssignR software; Figure S3: Average DBE, number of carbon, hydrogen, nitrogen, oxygen and sulfur of the MF common to the three SPE (“Common”), present only in SPE1, SPE2 or SPE3 (“only SPE1”, “only SPE2”, and “only SPE3”, respectively), common to SPE1 and SPE2 (“SPE1_SPE2”), to SPE1 and SPE3 (“SPE1_SPE3”) or SPE2 and SPE3 “SPE2_SPE3”. The error bar represents the standard deviation of the average intensity. The MF assignment was performed with DataAnalysis; Table S1: Microphysical and physico-chemical characterization of the cloud water sample; Table S2: List of internal recalibrants; Table S3: Parameters used for formula assignment; Table S4: High intensity MF in MFAssignR SPE2_SPE3. The column “Error (ppm)” reports the error associated to the formula assignment; “Composer” reports the fraction where the MF is present in Composer data treatment, with the associated error, and “DataAnalysis” the fraction in DataAnalysis data treatment, with the associated error.

## Sample Availability

Samples of the compounds are not available from the authors.
