# Supplementary material for: How Well Do We Handle the Sample Preparation, FT-ICR Mass Spectrometry Analysis, and Data Treatment of Atmospheric Waters?
Source: Molecules. 2022 Nov 12;27(22):7796. doi: 10.3390/molecules27227796 (PMC9692371; doi:10.3390/molecules27227796)
Supplement: Supplementary file 1 [file molecules-27-07796-s001.zip › molecules-1957776-supplementary.pdf]

Article

How Well Do We Handle the Sample Preparation, FT-ICR Mass Spectrometry Analysis, and Data Treatment of Atmospheric Waters?

Lucas Pailler [1], Pascal Renard [1], Edith Nicol [2], Laurent Deguillaume [1,3,*] and Angelica Bianco [1,*]

[1] Laboratoire de Météorologie Physique, UMR 6016, CNRS, Université Clermont Auvergne, 63178 Aubiere, France
[2] Laboratoire de Chimie Moléculaire, UMR 9168, CNRS, Ecole Polytechnique, 91128 Palaiseau, France
[3] Observatoire de Physique du Globe de Clermont-Ferrand, UMS 833, CNRS, Université Clermont Auvergne, 63178 Aubiere, France
* Correspondence: laurent.deguillaume@uca.fr (L.D.); a.bianco@opgc.fr (A.B.)

Table S1. Microphysical and physico-chemical characterization of the cloud water sample.

Measurement	Value	Methodology
pH	4.5	pHmeter
Cl^-	11.6 µM	Ion chromatography
NO_3^-	31.8 µM	Ion chromatography
SO_4^{2-}	12.9 µM	Ion chromatography
Na^+	16.5 µM	Ion chromatography
NH_4^+	33.5 µM	Ion chromatography
K^+	3.2 µM	Ion chromatography
Mg^{2+}	3.1 µM	Ion chromatography
Ca^{2+}	7.1 µM	Ion chromatography
HCHO	2.0 µM	Derivatization + fluorimetric determination
DOC	12.6 mgC L^{-1}	DOC analyser
H_2O_2	6.4 µM	Derivatization + fluorimetric determination

Table S2. List of internal recalibrants.

Ion formula	m/z
$C_7H_{11}O_5$	175.061197
$C_9H_{15}O_4$	187.097583
$C_8H_{13}O_5$	189.076847
$C_{13}H_{24}NO_3$	242.176167
$C_{16}H_{31}O_2$	255.232954
$C_{17}H_{27}O_3S$	311.168639
$C_{19}H_{31}O_3S$	339.199940
$C_{21}H_{34}NO_5$	380.244247
$C_{24}H_{34}N_4O_4$	451.328979
$C_{24}H_{42}NO_{10}S$	536.253491
$C_{44}H_{69}O_3S$	677.497291

Table S3. Parameters used for formula assignment.

Parameters for formula assignment	Composer	DataAnalysis	MFAssignR
Charge state	-1	-1	-1
Ion loss	H	H	H
Radicals allowed	No	No	No
Adducts	--	--	--
m/z range	100-1000 Da	100-1000 Da	100-1000 Da
DBE range	0-25	0-25	0-25
#C	1-70	1-70	1-70
#H	2-140	2-140	2-140
#N	1-25	1-25	1-25
#O	0-4	0-4	0-4
#S	0-1	0-1	0-1
m/z tolerance	0.5 ppm	0.5 ppm	0.5 ppm
DOM/NOM rules	Yes	No	Yes
De novo upper limit	300 Da	No	300 Da
Minimum de novo abundance	0.1%	--	0.1%

Table S4. High intensity MF in MFAssignR SPE2_SPE3. The column "Error (ppm)" reports the error associated to the formula assignment; "Composer" reports the fraction where the MF is present in Composer data treatment, with the associated error, and "DataAnalysis" the fraction in DataAnalysis data treatment, with the associated error.

MAF	Error (ppm)	Composer	Error (ppm)	DataAnalysis	Error (ppm)
$C_{45}H_{72}O_5S$	0.18	Not found	-	Common	-0.22
$C_{51}H_{76}O_5S$	-0.21	Not found	-	SPE1_SPE3	0.01
$C_{25}H_{39}N_3O_5$	0.23	Only SPE2	-0.23	Common	-0.13
$C_{19}H_{14}OS$	0.48	Common	-0.49	Common	-0.44

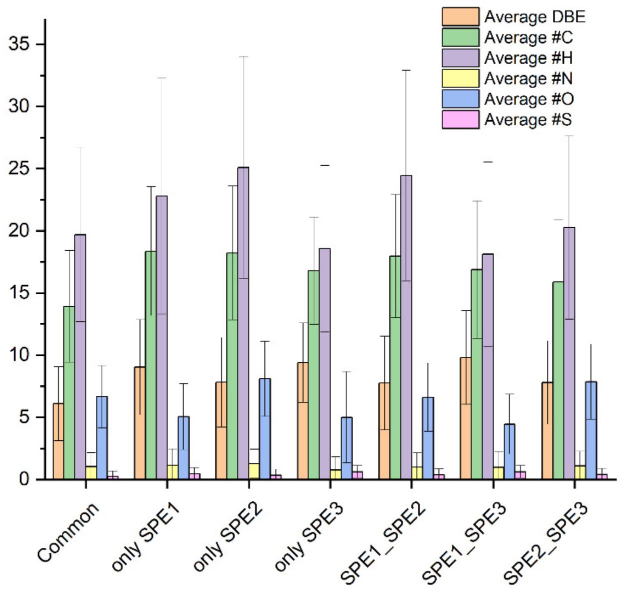

Figure S1. Average DBE, number of carbon, hydrogen, nitrogen, oxygen and sulfur of the MF common to the three SPE ("Common"), present only in SPE1, SPE2 or SPE3 ("only SPE1", "only SPE2", and "only SPE3", respectively), common to SPE1 and SPE2 ("SPE1_SPE2"), to SPE1 and SPE3 ("SPE1_SPE3") or SPE2 and SPE3 "SPE2_SPE3". The error bar represents the standard deviation of the average intensity. The MF assignment was performed with Composer software.

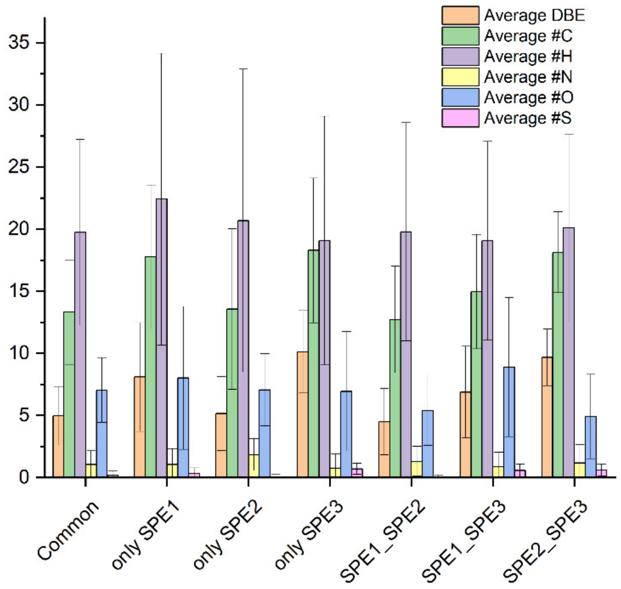

Figure S2. Average DBE, number of carbon, hydrogen, nitrogen, oxygen and sulfur of the MF common to the three SPE ("Common"), present only in SPE1, SPE2 or SPE3 ("only SPE1", "only SPE2", and "only SPE3", respectively), common to SPE1 and SPE2 ("SPE1_SPE2"), to SPE1 and SPE3 ("SPE1_SPE3") or SPE2 and SPE3 "SPE2_SPE3". The error bar represents the standard deviation of the average intensity. The MF assignment was performed with MFAssignR software.

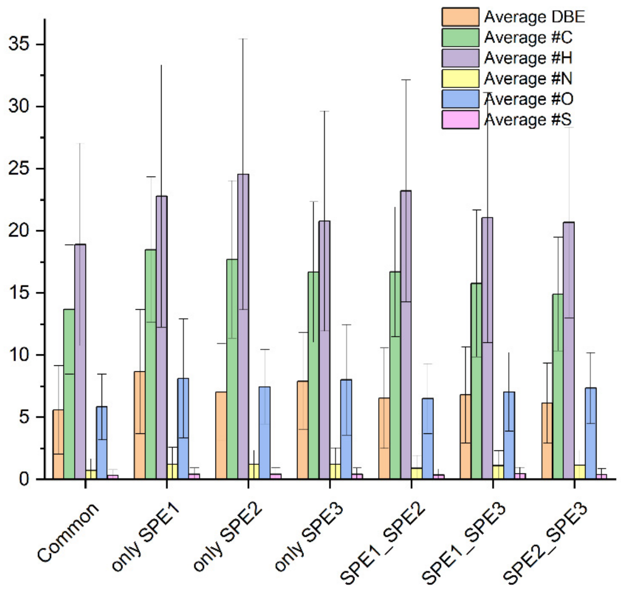

Figure S3. Average DBE, number of carbon, hydrogen, nitrogen, oxygen and sulfur of the MF common to the three SPE ("Common"), present only in SPE1, SPE2 or SPE3 ("only SPE1", "only SPE2", and "only SPE3", respectively), common to SPE1 and SPE2 ("SPE1_SPE2"), to SPE1 and SPE3 ("SPE1_SPE3") or SPE2 and SPE3 "SPE2_SPE3". The error bar represents the standard deviation of the average intensity. The MF assignment was performed with DataAnalysis.
